# Deciphering Direct and Indirect Effects of Neurokinin B and GnRH in the Brain-Pituitary Axis of Tilapia

**DOI:** 10.3389/fendo.2019.00469

**Published:** 2019-07-12

**Authors:** Naama Mizrahi, Chaim Gilon, Ishwar Atre, Satoshi Ogawa, Ishwar S. Parhar, Berta Levavi-Sivan

**Affiliations:** ^1^Faculty of Agriculture, Food and Environment, The Hebrew University, Rehovot, Israel; ^2^Institute of Chemistry, The Hebrew University of Jerusalem, Jerusalem, Israel; ^3^Jeffrey Cheah School of Medicine and Health Sciences, Brain Research Institute, Monash University Malaysia, Bandar Sunway, Malaysia

**Keywords:** tilapia, NKB, NKF, GnRH, gonadotropins, GH, FSH, LH

## Abstract

Neurokinin B (NKB) and its cognate receptor (NK3R) are emerging as important components of the neuroendocrine regulation of reproduction. Unlike mammalian *tac3*, which encodes only one mature peptide (namely NKB), two mature peptides are predicted for each *tac3* gene in fish and frogs. Therefore, it was designated as Neurokinin F (NKF). Hormone analogs with high and long-lasting biological activity are important tools for physiological and biological research; however, the availability of piscine-specific analogs is very limited. Therefore, we have developed specific NKB and NKF analogs based on the structure of the mammalian NKB analog–senktide. These analogs, specifically designed for longer half-lives by methylation of proteolysis sites, exhibited activity equal to those of the native NKB and NKF in short-term signal-transduction assays of tilapia NKB receptors. However, the analogs were found to be able to significantly increase the release of luteinizing hormone (LH), follicle stimulating hormone (FSH) and growth hormone (GH) in tilapia, as fast as 1 h after intraperitoneal (IP) injection. The impact of the analogs on LH and FSH secretion lasted longer compared to the effect of native peptides and salmon GnRH analog (sGnRHa). In addition, we harvested pituitaries 24 h post injection and measured LH, FSH and GH mRNA synthesis. Both analogs elevated mRNA levels of LH and GH, but only NKB analog increased FSH mRNA levels in the pituitary and all GnRH forms in the brain. NKB receptors were co-localized with all three types the GnRH neurons in tilapia brain *in situ*. We previously showed a direct effect of NKB at the pituitary level, and these new results suggest that the stronger impact of the NKB analog on GTH release is also due to an indirect effect through the activation of GnRH neurons. These results suggest that novel synthetic NKB analogs may serve as a tool for both research and agricultural purposes. Finally, the biological activity and regulatory role of NKB in tilapia brain and pituitary suggest that the NKB/NKBR system in fish is an important reproductive regulator in a similar way to the kisspeptin system in mammals.

## Introduction

Reproduction is an essential process that is regulated by numerous factors along the brain/hypothalamus-pituitary-gonad (HPG) axis. In teleost, there is a wide variety of reproductive strategies since they can be found in various environmental niches and different time regimes that elicit different modes of sexual maturation and behavior. Unlike other vertebrates, the fish anterior pituitary is innervated by neurons that synthesize a number of neuropeptides and neurotransmitters involved in the regulation of the release of both gonadotropins [GTHs; luteinizing hormone (LH) and follicle-stimulating hormone (FSH)], growth hormone (GH), and other pituitary hormones (1, 2). Among the neuropeptides released by these nerve endings are gonadotrophin-releasing hormones (GnRHs) and dopamine, which act as stimulatory and inhibitory factors on the release of GTHs (2).

Teleost fish possess two or three types of GnRH, whereas mammals have one to two. Like many teleosts, the Nile tilapia (*Oreochromis niloticus*) possesses three GnRH ligands, including seabream GnRH-1, chicken GnRH-2, and salmon GnRH-3. Neurons of the type 1 GnRH (sbGnRH-1) are expressed in ventral forebrain neurons and gather to enter a fiber tract (preoptico-hypophysial tract) that only innervates the pituitary (3). The type 2 GnRH (cGnRH-2) is conserved in all vertebrates and is present at the diencephalic-mesencephalic transition. Type 3 GnRH (sGnRH-3) is mainly produced in olfactory bulb/telencephalic neurons (4). During the past decade the neuropeptides kisspeptin (encoded by *Kiss1*) and neurokinin B (NKB, encoded by *tac3*) were identified as important regulators of GnRH synthesis and release in vertebrates [reviewed by Topaloglu and Kotan (5), Pinilla et al. (6), Parhar et al. (7)]. Kisspeptin, a neuropeptide belonging to the RFamide neuropeptide family, was found to be a key regulator of puberty and reproduction by regulating GnRH release. Humans and mice that possess mutation in the kiss1 receptor are infertile with hypogonadotropic hypogonadism (8, 9). Fish *kiss1* was first reported in zebrafish (*Danio rerio*) (10, 11) but in some teleost two genes were found encoding kisspeptin-like structure (12), nevertheless, some fish species, such as tilapia (13), red seabream (*Pagrus major)* (14) and more, express only the kiss2 type gene. Similar to its ligand, there are two or more kiss receptors (kissR) with different distribution patterns in teleost brains, suggesting their independent functions and potential roles (15). While kisspeptin is a key regulator in GnRH release in mammals (16), its role in teleosts remains controversial, especially since knockout of the two kisspeptins and two KissRs genes in zebrafish and medaka (*Oryzias latipes*) had no obvious effect on their reproductive capability (17, 18). Like kisspeptin, humans carrying loss-of-function mutations in neurokinin B [NKB; also known as tachykinin 3 (Tac3)] or in NKBR also display hypogonadotropic hypogonadism (19). The first indication that NKB is widely expressed in fish species and has a stimulatory reproductive effect was shown in zebrafish (20), goldfish (*Carassius auratus*) (21), European eel (*Anguilla Anguilla*) (22) and tilapia (23). Teleost have one or two *tac3* genes that encodes two mature peptides, NKB and NKB-related peptide (also called neurokinin F (NKF), and two tac3r genes (Tac3ra and Tac3rb) (20, 23). The synthetic zebrafish and tilapia NKBs activated Tac3 receptors via both PKC/Ca^2+^ and PKA/cAMP signal-transduction pathways *in vitro* (20, 23). Subsequent studies showed that LH cells, and to a lesser extent FSH cells, possess Tac3r, implying that NKB can act directly at the pituitary level (23), but very little is known regarding the effect of NKB at the level of the brain and its relationships with GnRH neurons.

The Nile tilapia is an important species in aquaculture and an emerging model system for laboratory studies in many fields. Several attributes give tilapia a significant advantage over the more common fish models like zebrafish and medaka, especially when performing physiological and behavioral studies, such as its inherent hardiness, ease of reproduction, and large size that enables researchers to perform multiple bleedings (24).

Hormone analogs with high and long-lasting biological activity are important tools for physiological and biological research; however, the availability of piscine-specific analogs is very limited. Such piscine-specific neuropeptide analogs that can enhance gonadotropin secretion will potentially serve as “next-generation” piscine reproduction inducers. In this study, we focused on new designs of piscine NKB and NKF analogs with the aim of improving bioavailability in order to investigate the regulatory role of the NKB system on the different GnRH neurons in the brain, in parallel to GTH and GH release and synthesis in the pituitary of tilapia.

## Materials and Methods

### Animals

Sexually mature female Nile tilapia [*O. niloticus;* BW 89.29 ± 32.93; Gonadosomatic Index (GSI) 0.24 ± 0.40%] were kept and bred in the fish facility unit at the Hebrew University in 500-L tanks at 26°C with 14L:10D photoperiod. Fish were fed daily with commercial fish pellets (Raanan Fish Feed, Miluot, Israel). GSI was calculated as gonad weight/BW × 100. All experimental procedures were approved by the Hebrew University administrative panel for laboratory animal care.

### NKB Peptides and Analogs Synthesis and Purification

Tilapia (ti) NKB (pyroglutaminated [p]-EMDDIFIGLM-NH2), tilapia NKF YNDLDYDSFVGLM-NH2), zebrafish (zf) NKBa (EMHDIFVGLM-NH2), zebrafish NKBb (STGINREAHLPFRPNMNDIFVGLLEMHDIFVGLM-NH2), and zebrafish NKF (YNDIDYDSFVGLM-NH2) were synthesized by GL Biochem. Peptides were synthesized by the automated solid-phase method by applying Fmoc active-ester chemistry. The crude peptides were purified by HPLC to >95% purity according to Hurevich et al. (25) and amidated at the carboxy terminus. The selective tiNKB analog (Succ-Asp-Ile-Phe-N(Me)Ile-Gly-Leu-Met-NH2), selective zfNKBa analog (Succ-Asp-Ile-Phe-N(Me)Val-Gly-Leu-Met-NH2), selective zfNKBb analog (Succ-Asp-Phe-N(Me)Val-Gly-Leu-Met-NH2), and selective NKF analog (Succ-Asp-Ser-Phe-N(Me)Val-Gly-Leu-Met-NH2), were synthesized by the standard Fmoc SPPS on Rink-Amide MBHA tentagel resin (loading 0.6 mM/gr). The crude peptides were purified by preparative RP-HPLC to >95% purity (25). The pure peptides had a single peak in analytical RP-HPLC with the expected mass determined by MS analysis. Peptides were dissolved to the desired concentration in fish saline (0.85% NaCl in DDW) for *in vivo* administration.

### Ligand Models

The basic homology models were built using Schrodinger Software (26) using the NMR structure of human Neurokinin B-'1p9f' (27) as a templet structure. The amino acids were then modified using the draw feature in Schrodinger.

### Receptor Signal Transduction Reporter Assays

Receptor transactivation assays were generally performed according to Biran et al. (10). Briefly, in order to study the signaling pathways of the novel fish NKB and NKF analogs, the entire coding sequence of tiTac3ra (GenBank accession no. KF471674), tiTac3rb (GenBank accession no. KF471675), zfTac3ra (GenBank accession no. JF317292) and zfTac3rb (GenBank accession no. JF317293) were inserted into pcDNA3.1 (Invitrogen). To differentiate between the protein kinase A (PKA) and PKC signal transduction pathways, we used a sensitive luciferase (Luc) reporter gene assay. We previously demonstrated that cAMP response element (CRE-Luc; Invitrogen) and serum response element (SRE-Luc; Invitrogen) reporter systems are useful tools for discriminating cAMP/PKA and Ca^2+^/PKC signaling pathways, respectively (10). The cDNA clone for human TAC3R was obtained from the Missouri S&T cDNA Resource Center (www.cdna.org). Three micrograms of each construct together with three micrograms of a luciferase reporter plasmid were transiently co-transfected into COS-7 cells. Forty-eight hours after transfection, cells were treated with tilapia or zebrafish NKB or NKF, huNKB, or their analogs at increasing doses. The hormone treatment and the subsequent measurement of luciferase activities were carried out as previously described (10, 20). EC50 values were calculated from dose response curves by means of computerized non-linear curve fitting on baseline-corrected (control) values using Prism version 6 software (GraphPad).

### Effects of Tilapia NKB, NKF, and Their Analogs *in vivo*

Adult female tilapia (BW 89.29 ± 32.93 g) were injected IP with saline, sGnRH analog (sGnRHa; 10 μg/kg BW; [D-Ala^6^,Pro^9^-Net]-mammalian GnRH; Bachem), tiNKB, tiNKF, or their analogs at 100 μg/kg BW (*n* = 8 fish per group). The fish were bled from the caudal blood vessels into heparinized syringes 1, 2, 4, 8, and 24 h after injection and blood was centrifuged (3,200 rpm for 30 min at 4°C) to obtain plasma samples, which were stored at −20°C until assayed. This time course is according to standard protocols used previously (28, 29) to test the effect of GnRH and other hypothalamic neuropeptides on circulating levels of LH, FSH, and GH in tilapia. Blood samples were collected from the caudal vasculature. Three independent experiments were carried out for the *in vivo* studies.

### ELISAs for the Measurement of Tilapia FSH, LH, and GH

Levels of LH, FSH, and GH in the plasma were measured by specific competitive ELISAs developed for tilapia (30, 31) based on recombinant (r)tiGTHs or rtiGH. Primary antisera was produced against rtLHβ, rtFSHβ, or rtGH (31), and rtLHβα (32), rtFSHβα (28) or rtGH was used for generating the standard curves. Sensitivity for the plasma measurements were 15.84 pg/ml for LH, 0.24 pg/ml for FSH and 35.0 pg/ml for GH. Inter-assay coefficient of variation (CV) was 14.8, 12.5, and 13%, while intra-assay CV was 7.2, 8, and 8% for LH, FSH and GH, respectively.

### RNA Extraction and Real Time PCR

Pituitaries and three parts of the brain [the olfactory bulbs and telencephalon (front brain), the optic tectum and diencephalon (mid brain), and the cerebellum (hind brain)] were harvested at the end of each *in vivo* experiment, snap-frozen in liquid nitrogen, and stored at −80°C until extraction. Total RNA was extracted by phenol-chloroform using TRIzol reagent (Invitrogen) and precipitated with ethanol, and 1 μg was used as a template for cDNA synthesis using Verso cDNA kit (Thermo Scientific, Waltham, MA). Real-time PCR analysis was performed according to Biran et al. (10, 20). Primer pair sequences and their corresponding efficiency and *R*^2^ values are listed in Table 1. The specificity of the amplification was tested at the end of the PCR by melting-curve analysis and sequencing the PCR products.

**Table 1:** Primers used for quantitative real-time PCR and *in situ* hybridization.

**Primer**	**Position**	**5′ to 3′ sequence**	**Slope**	***R*^**2**^**	**Application**
18S-F	660	GCACCACCACCCACAGAATC	−2.917	0.975	Real-time PCR
18S-R	897	CGACCATAAACGATGCCAACTAG			
βLH F	44	TGCTCCTTGCTCTGATGTTGA	−3.641	0.999	Real-time PCR
βLH R	226	CCTTGGTGATGCAGTGTCCAC			
βFSH F	174	CTGTCGCCCAAAGAACATCA	−3.543	0.998	Real-time PCR
βFSH R	424	AGGTCCCGCAGTCTGTGTTT			
tGH–F	151	TGCTCGCCCAGAGACTCTTC	−3.367	0.991	Real-time PCR
tGH–R	351	TGGGAAACTCCCAGGACTCA			
tiTacra –F	22	TCCAACATAACGACGAACCA			ISH
tiTacra -R	758	AGGGCAAACGTCACCACTAC			ISH
tiTacrb -F	208	GCTTTCAACACGCTCATCAA			ISH
tiTacrb -R	907	GCTTGAAACCAGCTCGAAAC			ISH

### *In situ* Hybridization and Immunofluorescence on Brain Cryosections

We used double-labeling of different GnRH types and Tac3r forms by combining immunofluorescence and *in situ* hybridization as described previously (33). Tilapia brains were harvested from sexually mature fish and fixed in 4% paraformaldehyde in PBS. After fixation, samples were cryoprotected in 20% sucrose and embedded in OCT compound (Sakura Finetechnical, Tokyo, Japan). Consecutive coronal sections (15 μm thick) were cut on a cryostat and thaw mounted onto 3-aminopropylsilane-coated glass slides (GAP1, #ISP105; GAP2, #ISP205, and GAP3, #ISP305) (13). *tac3r* mRNA expression was detected *in situ* with DIG-labeled riboprobes for tilapia *tac3ra* and *tac3rb* (Table 1) (33). DIG-labeled riboprobes were detected with a Tyramide Signal Amplification (TSA) Plus kit (PerkinElmer/NEN Life Science Products, Wellesley, MA) according to the manufacturer's instructions. Briefly, the hybridized brain sections were equilibrated in Tris-NaCl Tween (TNT) buffer containing 0.1 M Tris-HCl, pH 7.4, 0.15 M NaCl, and 0.05% Triton X-100. Slides were then incubated with a 1:500 dilution of peroxidase-conjugated anti-DIG antibody (Roche Applied Science) in TNT buffer with 1% normal goat serum at room temperature for 2 h. Color development was carried out with a 1:50 dilution of reconstituted fluorescent tyramide to the diluent of Plus amplification (PerkinElmer/New Life Science Products) for a period of 5 min.

Following *in situ* hybridization for either *tac3ra* or *tac3rb*, the three GnRH types (GnRH1, GnRH2, and GnRH3) were detected by rabbit anti-tilapia GnRH antibodies against their respective GnRH associated peptide (GAP) sequence (GAP1, #ISP105; GAP2, #ISP205, and GAP3, #ISP305). TSA-labeled sections of three brain regions containing respective GnRH types were rinsed in 0.01 M PBS and then incubated with tilapia GAP1, GAP2, or GAP3 antiserum (1:500) overnight at 4°C, then with Alexa Fluor 594-labeled anti-rabbit IgG (1:500; Invitrogen) for 2 h at room temperature. Separate images were captured by using a fluorescence microscope (ECLIPS 90i; Nikon Instruments) that was attached to a digital cooled CCD camera (DMX1200; Nikon) with appropriate excitation filters for fluorescein (Tac3ra and Tac3rb) and Alexa Fluor 594 (GAP types), and computer software (NIS Elements D3.0; Nikon) was used to superimpose the two images. The red channel was then converted to magenta, and brightness and contrast adjustments were made in Adobe Photoshop CC (Adobe Systems, San Jose, CA).

### Statistical Analysis

The results are presented as the mean±SEM. Two-way ANOVA for the ELISA assays and one-way ANOVA for the qPCR results were used to compare mean values of the *in vivo* experiments. This was followed by an a posteriori Dunnet test (compare to control) only when ANOVA revealed the presence of statistical significant differences between groups, using JMP version7 software.

## Results

### Novel Neurokinin B and Neurokinin F Synthetic Analogs

Following the findings that NKB is an important regulator of the reproductive axis in fish (20, 23, 34) we created analogs on the basis of the well-established human NKB analog senktide (Selective Neurokinin B receptor peptide) (35, 36). Sequences of NKB and NKF were shortened to the seven C-terminal amino acids (aa), an N-terminal Succinyl was added, and the valine (V) aa in the signature FVGLM sequence was methylated. tiNKB analog (Succ-Asp-Ile-Phe-N(Me)Ile-Gly-Leu-Met-NH_2_), zfNKBa analog (Succ-Asp-Ile-Phe-N(Me)Val-Gly-Leu-Met-NH_2_), zfNKBb analog (Succ-Asp-Phe-N(Me)Val-Gly-Leu-Met-NH_2_), and NKF analog Succ-Asp-Ser-Phe-N(Me)Val-Gly-Leu-Met-NH_2_,.

**Figure 1:**
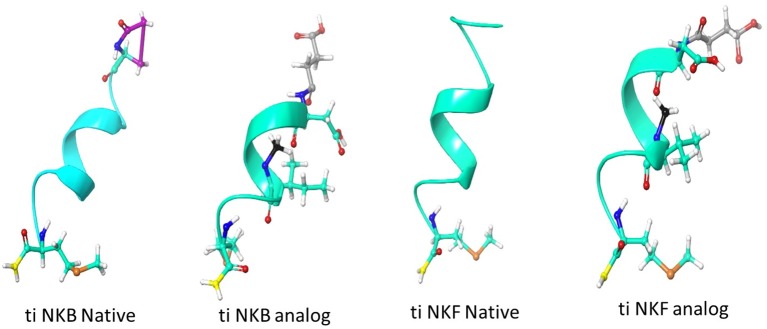
Structural model of human and tilapia NKBs and their analogs represented as cartoon ribbons. The structural models were predicted using human NKB (PDBID 1p9f) as a template. The amino acid modifications were then added using draw feature in Schrodinger, the succinylation is shown in gray, N-methyl is shown in black, amidation is shown in yellow and pyroglutamination is shown in pink.

We first aimed to compare the predicted structural models of the known human NKB (PDB ID 1p9f), the native piscine peptides and the novel analogs. The predicted models were observed to be α- helix loop-rich peptides pointing in one direction toward the amide NH groups. The hydrogen bonds between the residues were formed every 4th amino acid (5.4 Å). These structures displayed a high structural similarity to their human and zebrafish homologs (20) and superposition with the human template (27), suggesting similar function (Figure 1).

### Signal-Transduction Activity of NKB Analogs

**Figure 2:**
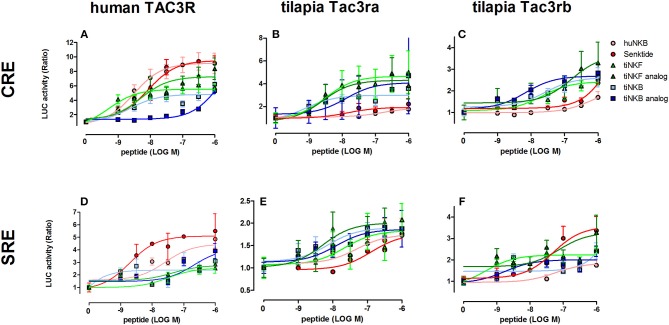
Tilapia NKB peptides and analogs induced transcriptional activity in CRE- and SRE-Luciferase reporters. COS-7 cells were co-transfected with human TAC3R **(A,D)**, tilapia (ti) Tac3ra **(B,E)**, and tilapia Tac3rb **(C,F)** together with a reporter plasmid. Cells were treated with various concentrations of human and tilapia NKB peptides and their analogs. The data are expressed as the change in luciferase activity over basal and are from a single experiment representative of a total of 3 such experiments. Each point was determined in triplicate and is shown as a mean ± SEM.

**Figure 3:**
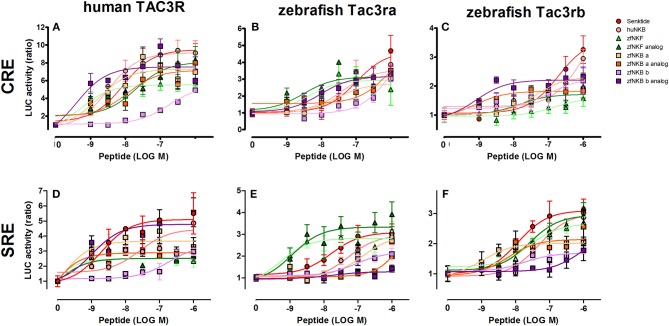
Zebrafish NKB peptides and analogs induced transcriptional activity in CRE- and SRE-Luciferase reporters. COS-7 cells were co-transfected with human TAC3R **(A,D)**, zebrafish Tac3ra **(B,E)**, and zebrafish Tac3rb **(C,F)** together with a reporter plasmid. Cells were treated with various concentrations of human and zebrafish NKB peptides and their analogs. The data are expressed as the change in luciferase activity over basal and are from a single experiment representative of a total of 3 such experiments. Each point was determined in triplicate and is shown as a mean ± SEM.

We first aimed to analyze the potency, selectivity, and signal transduction pathways of the novel NKB and NKF analogs to activate NKBRs *in vitro* using COS-7 cells. We previously showed the specificity of the reporter serum responsive element (SRE)-Luc and cAMP responsive element (CRE)-Luc to activation of PKC/Ca^2+^ and PKA/cAMP signal transduction pathways, respectively (10). Signal transduction analysis showed that each of the novel analogs is equally or more potent than its corresponding natural ligand. The EC_50_ values of NKBs for each receptor are summarized in Tables 2, 3. Tilapia and zebrafish NKB receptors were activated by the human, tilapia and zebrafish NKBs dose-dependently in both signal transduction pathways. Human TAC3R was activated by all ligands (human, tilapia, and zebrafish), while the human ligand was less effective in activating the piscine receptors (Figures 2, 3). Analogs for tiNKF (EC_50_ = 0.51 nM, Figure 2A) and zfNKBb (EC_50_ = 0.39 nM, Figure 3A) were more efficient in activation of the PKA/cAMP pathway, whereas analogs for tiNKB (EC_50_ = 0.022 nM, Figure 2D) and zfNKBa (0.03 nM, Figure 3D) were more efficient in activation of the PKC/ Ca^2+^ pathway. For most of the NKB peptides tested, tiTc3rb was stimulated at lower efficiency than tiTac3ra in both signal transduction pathways. There were no major differences in activation between the native peptides and their analogs for tiTac3ra. Although, the PKA pathway was more efficient than the PKC pathway, tiNKB was the most efficient peptide along the PKA pathway and the tiNKF was the most efficient in the PKC pathway (EC_50_ = 0.8 and 0.2 nM, respectively; Figures 2B,E). zfNKBb and NKF analog increases luciferase activity in the PKA pathway transfected together with zfTac3ra (EC_50_ = 0.94 and 0.47 nM„ respectively; Figure 3B). Along PKC pathway the zfNKF was the most efficient (EC_50_ = 0.51 nM; Figure 3E). Most of the zebrafish ligands increase luciferase activity in higher efficiency in the PKA pathway than the PKC pathway in the zfTac3rb transfected cells. Both analogs and native peptides have similar effect on the zfTac3 receptors, same as the tiTac3 receptors.

**Table 2:** EC_50_ values (nM) of human and tilapia NKB receptors.

	**huTAC3R (NK3R)**		**tiTac3ra**		**tiTac3rb**	
	**CRE**	**SRE**	**CRE**	**SRE**	**CRE**	**SRE**
huNKB	3.06 ± 0.07	26.06 ± 0.42	163.4 ± 0.36	48.3 ± 0.32	676.1 ± 0.4	57.72 ± 0.58
Senktide	8.46 ± 0.19	1.94 ± 0.52	14.06 ± 0.61	128.2 ± 035	3541 ± 1.47	50.16 ± 0.17
tiNKB	3.38 ± 0.42	0.02 ± 0.38	0.81 ± 0.47	7.41 ± 0.44	26.48 ± 0.27	–
tiNKB analog	–	210.2 ± 1.18	12.94 ± 0.36	12.32 ± 0.29	6.88 ± 0.29	1.73 ± 0.46
tiNKF	0.51 ± 0.24	32.58 ± 0.05	3.17 ± 0.18	0.24 ± 0.77	2.13 ± 0.42	0.46 ± 0.49
NKF analog	18.23 ± 0.21	43.23 ± 1.062	2.57 ± 0.33	1.73 ± 0.46	160.6 ± 0.42	74.89 ± 0.66

*cAMP responsive element (CRE)-Luc was used as a reporter of PKA activation; Serum responsive element (SRE)-Luc was used as a reporter of PKC activation. Results are expressed as mean ± SEM*.

**Table 3:** EC_50_ values (nM) of human and zebrafish NKB receptors.

	**huTAC3R (NK3R)**		**zfTac3ra**		**zfTac3rb**	
	**CRE**	**SRE**	**CRE**	**SRE**	**CRE**	**SRE**
huNKB	3.06 ± 0.07	26.06 ± 0.42	40.74 ± 0.39	51.51 ± 0.1	927.3 ± 0.36	44.9 ± 0.16
Senktide	8.46 ± 0.19	1.94 ± 0.52	14.06 ± 0.61	128.2 ± 035	3541 ± 1.47	50.16 ± 0.17
zfNKBa	1.71 ± 0.32	0.11 ± 0.3	26.48 ± 0.38	139.6 ± 0.38	41.37 ± 0.18	1.105 ± 0.13
zfNKBa analog	11.47 ± 0.4	0.03 ± 0.15	873.2 ± 0.64	7.876 ± 1.89	0.781 ± 0.15	5.795 ± 0.52
zfNKBb	17.4 ± 0.15	18.67 ± 0.25	0.948 ± 0.43	84.98 ± 0.31	101.4 ± 0.37	17.88 ± 0.43
zfNKBb analog	0.392 ± 0.53	1.01 ± 0.49	9.362 ± 0.15	18.97 ± 1.01	0.823 ± 0.34	893.2 ± 0.42
zfNKF	1.25 ± 4.36	0.18 ± 0.52	5.47 ± 0.43	0.51 ± 0.36	0.032 ± 0.44	25.51 ± 0.23
NKF analog	1.3 ± 7.33	0.71 ± 1.41	0.474 ± 0.56	1.15 ± 0.40	2.688 ± 0.38	25.74 ± 0.24

*cAMP responsive element (CRE)-Luc was used as a reporter of PKA activation; Serum responsive element (SRE)-Luc was used as a reporter of PKC activation. Results are expressed as mean ± SEM*.

### Effect of Tilapia NKB and NKF Analogs on GTH and GH Release and Expression *in vivo*

**Figure 4:**
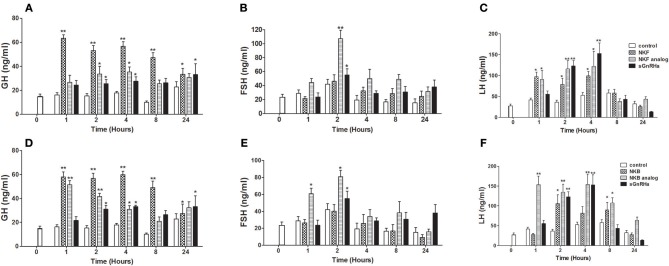
*In vivo* effect of tilapia NKB or NKF and their analogs on GH **(A,D)**, FSH **(B,E)**, and LH **(C,F)** plasma levels. Female tilapia were injected with native NKB or NKF peptides, analogs (100 μg/kg BW), or sGnRHa (10 μg/kg BW) at time 0; saline-injected fish served as controls. Blood was sampled at 1, 2, 4, 8, and 24 h after injection. Plasma GH, FSH, and LH values were analyzed by specific ELISA (Mean±SEM; *n* = 8 fish per group). Columns marked by asterisks significantly differ from basal: *p < 0.05; **p < 0.01 (two-way ANOVA followed by Dunnett).

We next sought to compare the effects of native NKB and NKB analogs on mRNA synthesis and release of the GTHs and GH in tilapia *in vivo*. In a similar manner to the response to the native peptides and sGnRHa, NKF analog increased plasma LH as early as 1 h and plasma FSH 2 h post-injection (Figures 4B,C). Both FSH and LH secretion was increased as early as 1 h post-injection of NKB analog (Figures 4E,F), and LH remained significantly high (*p* ≤ *0.01*) up to 12 h. Plasma GH increased in response to all treatments 1 or 2 h post-injection (Figures 4A,D).

**Figure 5:**
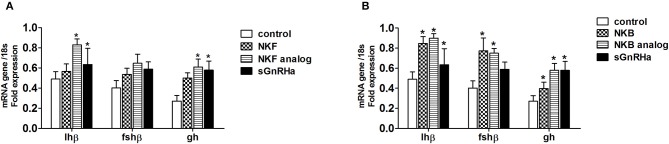
*In vivo* effect of tilapia NKF **(A)** or NKB **(B)** and their analogs on *gh, fsh* and *lh* mRNA expression. Female tilapia were injected with native peptides, analogs (100 μg/kg BW), or sGnRHa (10 μg/kg BW) at time 0; saline-injected fish served as controls. Pituitary samples were collected 24 h post injection for RNA extraction. Columns marked by asterisks significantly differ from basal: *p < 0.05; (one-way ANOVA followed by Dunnett).

After 24 h pituitaries were collected for RNA extraction and relative LH, FSH and GH mRNA expression was analyzed. NKF analog significantly (*p* ≤ *0.05*) increased LHβ and GH mRNA expression levels relative to the control (Figure 5A), while the native peptide of NKF did not. Both NKB and its analog increased the mRNA expression of LHβ, FSHβ, and GH in the pituitary compared to the control (Figure 5B).

### Effect of NKB Analogs on GnRH Expression *in vivo*

**Figure 6:**
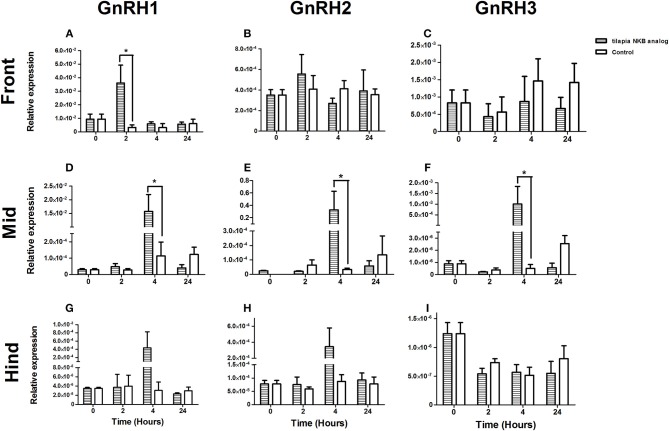
mRNA levels of GnRH1 **(A,D,G)**, GnRH2 **(B,E,H)**, and GnRH3 **(C,F,I**) in specific parts of the tilapia brain (front, mid, and hind) in response to IP injection of tiNKB analog (100 μg/kg BW). The relative abundance of the mRNAs was normalized to the amount of 18s by the ΔΔCT cycle method. Results are expressed as mean ± SEM (*n* = 4–5 fish/group). *p < 0.05 one-way ANOVA followed by Dunnett.

One of the suggested modes of action of NKB in mammals is through its direct effect on GnRH neurons (37, 38). To gain insight into the effect of NKB on GnRH expression in tilapia brain, we injected NKB analog (100 μg/kg BW) into sexually mature female tilapia and measured GnRH mRNA expression in specific areas of the brain. In tilapia, sbGnRH1 is the hypophysiotrophic form produced by neurons of the hypothalamic-preoptic area and project directly to the pituitary (39). GnRH1 mRNA expression significantly increased in the forebrain in response to NKB analog 2 h post-injection (Figure 6A), after 4 h in the midbrain (Figure 6D). Expression of GnRH2 (chicken GnRH-II) mRNA increased in the mid brain 4 h post-injection (Figure 6E), and remained unchanged in the forebrain and hindbrain (Figures 6B,H). Levels of GnRH1 mRNA did not significantly increase in the hind brain (Figure 6G). In tilapia, GnRH3 (salmon GnRH) cell bodies were found to be localized in the transitional area between the olfactory bulb and the telencephalon (40). In accordance, we found that GnRH3 was highly expressed in the forebrain of controls (Figure 6C), but the expression did not change in response to NKB analog. In the mid brain, GnRH3 expression increased in response to NKB analog (Figure 6F), but the expression was lower than that of the forebrain. No change in response to NKB was found in the hind brain (Figure 6I).

### Co-localization of tiTac3RA or tiTac3RB on GnRH Neurons

**Figure 7:**
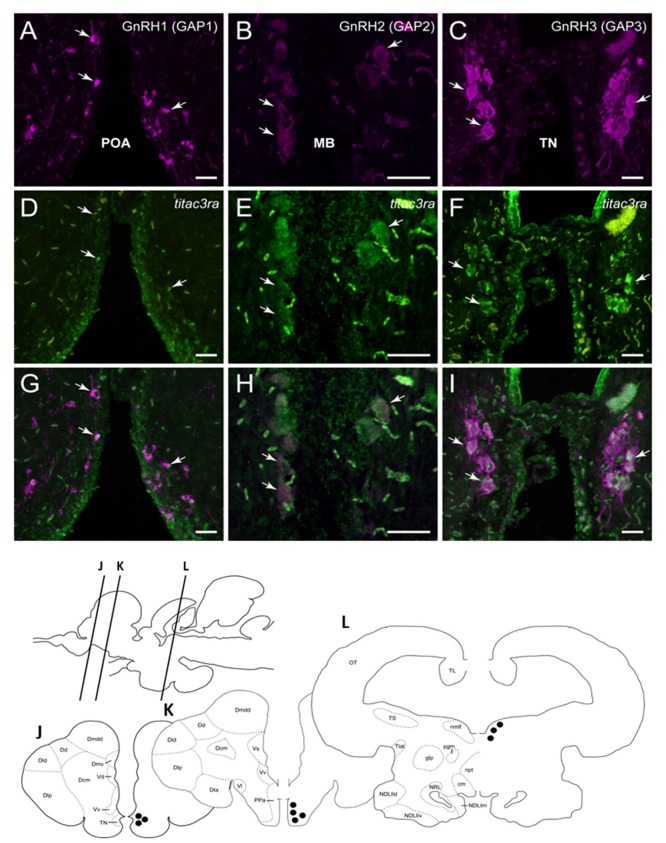
Tilapia *tac*3ra is expressed in GnRH1 and GnRH3, but not in GnRH2, neurons in tilapia brain. Double-labeled ISH-IF of *tac*3ra mRNA (green, **D,E,F**) and GnRH1 (red, **A,D,G**), GnRH2 (red, **B,E,H**), and GnRH3 (red, **C,F,I**) in the tilapia brain. Scale bar, 50 μm. Schematic representation of localization of three GnRH-associated peptides (GAP1, GAP2, and GAP3)-immunoreactive (-ir) neurons in the brain of tilapia. Lines on a schematic sagittal drawing view of the tilapia brain indicate levels of transverse brain sections **(J–L)** shown below. Black dots indicate the location of GnRH1, GnRH2 and GnRH3 (GAP1, GAP2, and GAP3-ir) neurons. GAP3-ir cells in the terminal nerve ganglia at the caudal-most olfactory bulbs (TN, **J**); GAP1-ir cells in the anterior part of the parvocellular preoptic nucleus (PPa, **K**) and GAP2-ir cells in the midbrain nucleus of the medial longitudinal fasciculus (nmlf, **L**). cm, mammillary body; Dcm, medial subdivision of central part of the dorsal telencephalon; Dd, dorsal part of the dorsal telencephalon; Dla, anterior subdivision of lateral part of the dorsal telencephalon; Dld, dorsal subdivision of lateral part of the dorsal telencephalon; Dlp, posterior subdivision of lateral part of the dorsal telencephalon; Dmdd, dorsal part of dorsal subdivision of medial part of the dorsal telencephalon; Dmv, ventral subdivision of medial part of the dorsal telencephalon; glp, posterior part of the glomerular nucleus; NDLlld, dorsal subdivision of lateral part of the diffuse nucleus of the inferior lobe; NDLllv, ventral subdivision of lateral part of the diffuse nucleus of the inferior lobe; NDLlm, medial part of the diffuse nucleus of the inferior lobe; npt, posterior tuberal nucleus; NRL, nucleus of the lateral recess; OT, optic tectum; pgm, medial preglomerular nucleus; TL, longitudinal torus; Tlat, nucleus of the lateral torus; TS, semicircular torus; Vd, dorsal part of the ventral telencephalon; Vl, lateral part of the ventral telencephalon; Vv, ventral part of the ventral telencephalon.

**Figure 8:**
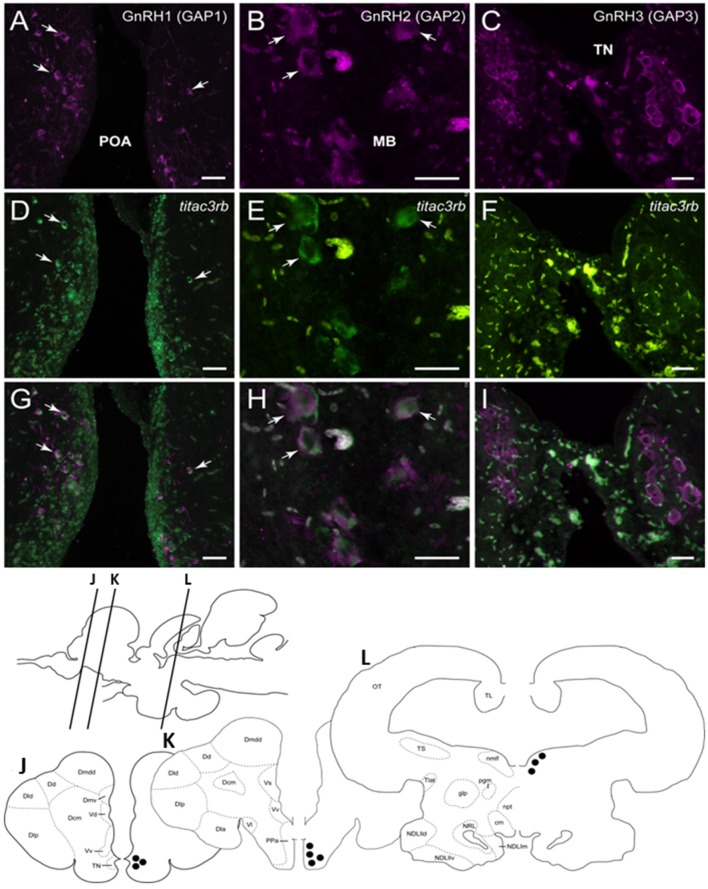
Tilapia *tac*3rb is expressed in GnRH1 and GnRH,2 but not in GnRH3, neurons in tilapia brain. Double-labeled ISH-IF of *tac*3rb mRNA (green, **D,E,F**) and GnRH1 (red, **A,D,G**), GnRH2 (red, **B,E,H**), and GnRH3 (red, **C,F,I**) in the tilapia brain. Scale bar, 50 μm. Schematic representation of localization of three gonadotropin-releasing hormone (GnRH)-associated peptides (GAP1, GAP2, and GAP3)-immunoreactive (-ir) neurons in the brain of tilapia. Lines on a schematic sagittal drawing view of the tilapia brain indicate levels of transverse brain sections **(J–L)** shown below. Black dots indicate the location of GnRH1, GnRH2, and GnRH3 (GAP1, GAP2, and GAP3-ir) neurons. GAP3-ir cells in the terminal nerve ganglia at the caudal-most olfactory bulbs (TN, **J**); GAP1-ir cells in the anterior part of the parvocellular preoptic nucleus (PPa, **K**) and GAP2-ir cells in the midbrain nucleus of the medial longitudinal fasciculus (nmlf, **L**). Other abbreviations are listed in the legend of Figure 7.

Since we showed an effect of NKB analog on GnRH synthesis *in vivo*, we tested whether or not this is through a direct action of NKB on the various GnRH neurons by looking for co-localization of the different Tac3Rs on the various GnRH neurons via *in situ* hybridization (ISH) and fluorescence immunohistochemistry (IF) methodologies. GnRH3 neurons co-localized with *tac3ra* (Figures 7C,F,I), but not *tac3rb* mRNA (Figures 8C,F,I) in the telencephalon. GnRH1 co-localized with both of the Tac3 receptors, but to a much lesser extent than the other two GnRH forms (Figures 7A,D,G, 8A,D,G). Finally, GnRH2 neurons co-localized with ti*tac3rb* but not ti*tac3ra* (Figures 7B,E,H, 8B,E,H).

## Discussion

Neurokinin B (NKB) is already established as a key player in the regulation of fish reproduction (20, 23). The tac3 genes and their receptors were first identified in zebrafish, and it was predicted by cleavage analysis that preprotachykinin 3 encodes two mature peptides produced from each tac3 gene in many fish species (20, 34, 41). One of the peptides that was generated by the processing of the Tac3 precursors was similar to the mammalian NKB, while the second related peptide was termed NKF due to its existence only in fish and frogs (20). Treatment with NKB peptides caused an increase in both FSH and LH synthesis and release in zebrafish (20), tilapia (23), goldfish (21), striped bass (*Morone saxatilis*) (42) and orange spotted grouper (*Epinephelus coioides*) (43) while in the European eel, human NKB was found to inhibit the expression of lhβ (22). Moreover, NKB and NKF bound and activated Tac3 receptors of zebrafish (20) and tilapia (23). We have developed specific NKB analogs to further study the mode of action of NKB and GnRH in fish, and showed that NKBs stimulated the expression and release of LH, FSH, and GH. Additionally, NKB increased the expression of each GnRH types in the tilapia brain, and NKB receptors co-localized with all GnRH neuronal forms.

### Transactivation of Fish NKB Receptors by NKBs and Their Analogs

Binding of NKB to its receptor, Tac3r, can be relayed by either increase in intracellular Ca^2+^ concentration through inositol phospholipid hydrolysis and activation of PKC, or intracellular cAMP levels through the activation of adenylate cyclase and PKA (44, 45). The pharmacological profiles of Tac3ra and Tac3rb already confirmed that both receptors have functional activities in the cAMP/PKA and Ca^2+^/PKC pathways in tilapia (23) and zebrafish (20, 41). In the current study, we aimed to analyze the abilities of the novel NKB and NKF analogs to activate Tac3rs *in vitro*. Senktide is the most active and selective mammalian NK3R agonist discovered thus far (35). The design of the fish NKB agonists was based on the development of senktide, namely by omitting the N-terminal sequence up to Asp^2^ (D) and replacing Val or Ile with N-Methyl Val or N-Methyl Ile. It was also shown that methylation of Phe^8^ and replacement of the three amino terminal amino acids with a succinyl moiety imposed selectivity to the Tac3r (35).

We have shown before that the cAMP/PKA pathway was preferred by the Tac3rs in response to the native peptide for tilapia (23) and zebrafish (20). In a similar way, under the current investigation, the cAMP/PKA pathway showed increased activation by the NKB and NKF agonists. Upon binding to the Tac3 receptors, the tilapia and the zebrafish NKB/NKF analogs were as efficient as the native peptides in increasing luciferase activity. tiTac3rb showed higher SRE- and CRE-luciferase activity for NKB analogs than the native peptides, whereas tiTac3ra had higher luciferase activity for both pathways with the native peptides over the analogs. The native peptide of NKF and its analog induced similar CRE-luciferase activity at tiTac3ra, but native NKF had slightly higher activation of the PKC pathway than the agonist. Native NKF was a more potent inducer of both PKA and PKC pathways through tiTac3rb than its analog. zfTac3ra. In the zfTac3ra the NKF analog has the higher luciferase activity, but with a similar response with the zfNKBb native peptide in the cAMP/PKA pathway. In the zfTac3rb transfected cells, the CRE-Luc pathway was more efficient when activated by most of the ligands except for the native peptides of zebrafish NKB. Similar results were reported for the human NK3R agonist, senktide, with native hNKB, which have similar abilities to activate in CHO (46) and guinea pig ileum (35) cell lines.

### NKB Analogs Increase Pituitary mRNA Expression and Release of LH, FSH, and GH

Administration of NKB or senktide in mammals have resulted in disparate effects on LH secretion depending on the animal model and gonadal maturity. Initial studies showed that intraventricular injections of senktide significantly decreased LH levels in ovariectomized rats treated with very low levels of estradiol (47), and senktide stimulated LH secretion in the ewe during the follicular phase, but not during the luteal phase (48). Stimulation of LH secretion was also observed in prepubertal rhesus monkeys after intravenous infusion of either NKB or senktide (49), while a single injection of senktide also increases LH and FSH levels in rats (50).

One of the advantages of senktide as an analog is its metabolic stability when compared to other NKB analogs, which were rapidly degraded by proteases in the brain, pituitary, and blood (36). The piscine NKB analogs, reported here, were developed based on senktide, so we hypothesized a prolonged effect on LH and FSH release, relative to the native peptides, due to decreased metabolism and clearance of the analog from circulation. In this report we investigated the effects of tilapia NKB and NKF analogs on LH, FSH, and GH synthesis and release. The results show that LH and FSH levels were increased in the plasma as fast as 1 h post injection with NKB analog, while the native NKB started to increase LH and FSH plasma levels only 2 h after the injection. NKB analogs induced an increase in plasma LH and FSH similar to the sGnRHa treatment, suggesting the importance of the NKB system in reproduction. NKB analog treatment not only shortened the initiation of the response, but also prolonged the duration of LH release up to 8 h post injection, compared to the native NKB and the sGnRHa treatments, which increased LH levels in the plasma up to 4 h only. Injection of NKF analog caused the same effect on LH, but not FSH, release.

In addition to the release of both gonadotropins, we also found significant GH release in response to NKB, NKF, and their analogs. The GH increase in response to both tiNKB and tiNKF was as fast as 1 h post injection, while the prolonged effect on GH release occurs in reaction to the native NKB and NKF treatment, not their analogs. There are several lines of evidence suggesting a link between GH and reproduction in tilapia: GnRH stimulates the release of GH both *in vivo* and *in vitro* (31). Overexpression of Gh in tilapia leads to disruption of reproduction, although it does not inhibit it completely (51), while gonadectomy significantly delayed growth compared to the shame-operated control fish (52).

It was shown that in carp (*Cyprinus carpio*) pituitary cells, NKB and NKF treatment up-regulated prolactin (PRL) and somatolactin (SL) α secretion, and synthesis, without altering GH, LHβ, FSHβ, GtHα, TSHβ, SLβ, and pro-opiomelanocortin (POMC) transcript expression (53). Unquestionably, further studies are needed to investigate the connection between the axes leading to growth and reproduction in fish.

### NKB Analogs Regulate GnRH Expression in Tilapia Brain

The demonstration of two or three forms of GnRH in individual vertebrate species, together with their wide anatomical distribution and effects on diverse cells and tissues, indicate that GnRHs have been coopted for multiple functions throughout evolution (54). The effect of GnRH on the regulation of FSH, LH, and GH secretion is well-established [reviewed by Levavi-Sivan et al. (2)] Marchant et al. was the first to demonstrate that GnRH stimulated GH release in goldfish (55). This was later confirmed in common carp (56), tilapia (57), rainbow trout (*Oncorhynchus mykiss*) (58), grass carp (*Ctenopharyngodon idella*) (59), and steelhead trout (*Oncorhynchus mykiss*), (60). Recently, Chen et al. identified co-localization of GnRHR2 on somatotrophs in an eel, *Monopterus albus* (61). Studies on the biological activity of GnRH agonists and antagonists indicate that GnRH receptors on somatotrophs are functionally distinct from those on gonadotrophs [reviewed in Dai et al. (62)]. Regulation of GnRH in fish pituitary cells is not limited to gonadotrophs and somatotrophs. All three forms of GnRH (sbGnRH1, cGnRH2, and sGnRH3) increased serum PRL in tilapia (*Oreochromis mossambicus*) *in vitro* (63). GnRH receptors co-localized with GtHs, GH, somatolactin, and PRL cells in pejerrey *(Odontesthes bonariensis)* using a pituitary primary cell culture system (64). In mammals, physiological secretion of GnRH occurs in a pulsatile manner. However, sustained exposure to GnRH is known to desensitize gonadotropin secretion (65). Desensitization of gonadotropes after continuous GnRH exposure is evident in some fishes (66) but absent in others (2, 67–69). A bolus of senktide during the last 3 h of continuous analog administration failed to elicit GnRH release, thus confirming desensitization of NK3R in agonadal juvenile male rhesus monkey *(Macaca mulatta)* (70). Although desensitization of the teleost NKB receptor has never been studied, the long term effect of tilapia NKB analog on GTH release may suggest that, like the tilapia GnRH type 3 receptor, receptor desensitization does not occur in tiTac3r's in response to tilapia NKB analog. In mammals, kisspeptin, neurokinin B and dynorphin neurons are all expressed in the same set of neurons (designated as KNDy neurons) and were found to regulate the generation of pulsatile GnRH release [reviewed by Lehman et al. (71)]. Unlike their mammalian orthologs, no co-localization of *tac3* and *kiss* mRNA was observed in zebrafish (34), showing the absence of KNDy neurons in fish. The lack of anatomical evidence for a complete neuronal network of kisspeptin, NKB, GnRH, and their cognate receptors is the main problem in elucidating the functions and relationship between these important players in fish reproduction. However, it was shown in tilapia that all three types of GnRH neurons expressed kissr2 mRNA (72). In another cichlid fish (*Astatotilapia burtoni*) it was shown by double-labeled ISH analysis that GnRH1 and GnRH3 neurons were co-expressed with kissr2 transcripts (73). Furthermore, *in vivo* treatment with Kiss2 modulated the reproductive axis and gonadal development in both males and females tilapia (23, 74). NK3R co-expression with GnRH neurons is not as robust as kissR/GnRH co-expression in mammals. In a similar way it was shown that NKB directly induce GnRH secretion from NK3R-expressing GnRH neurons of the rat median eminence (37), and GT1-7, a mouse hypothalamic GnRH neuronal cell line, express NK3R and respond to acute senktide treatment with increased GnRH secretion (38).

In this study, we found that the mRNA expression of all three GnRH types were increased 2–4 h after a single IP injection of tiNKB analog. Moreover, our results showed that all three types of GnRH neurons in the tilapia brain co-express at least one of the NKB receptors, suggesting a direct regulation of NKB on GnRH secretion resulting in increased gonadotropin secretion. Moreover, administration of NKB and NKF reduced *kiss1* and *kiss2* gene expression in the brain and pituitary of the male striped bass and hypothalamic Tac3 neurons innervated proximal Kiss2 neurons in the dorsal and ventral NRL, which in turn express Tac3r (42). Despite the lack of KNDy neurons in teleosts, a neuronal network of these neurohormones and receptors exists in modern fish and, through some unknown mechanism, integrates multiple inputs and regulates reproduction. The integral regulation of the reproductive axis by hypothalamic neuropeptides still requires further investigation.

In summary, hormone analogs with high and long-lasting biological activity serve as highly important tools for biological research. Such piscine-specific neuropeptide analogs are able to enhance gonadotropin secretion and can potentially serve as piscine reproduction inducers. The novel NKB analogs, specifically designed to have a longer half-life *in vivo*, exhibited activity equal to those of the native NKB and NKF in signal-transduction assays with NKB receptors. However, they were able to induce the release of FSH, LH, and GH in tilapia *in vivo* as fast as 1 h after IP injection with prolonged effects compared to the native peptides and sGnRHa, which elicited slower responses. Moreover, GTH and GH mRNA expression also increased in response to NKB and NKF analogs. NKB analogs induced mRNA synthesis of all three GnRH types in the brain, and NKB receptors were co-expressed in all three GnRH neurons in different regions of the brain, suggesting that besides the known direct effect of NKB on NKB receptors at the pituitary level (23), there is an indirect effect through secretion of GnRH that ends with increased secretion of gonadotropins. The biological activity and regulatory role of NKB in the tilapia brain and pituitary suggest that the NKB/NKBR system in fish is an important reproductive regulator in a similar way as the kisspeptin system is in mammals.

## Data Availability

All datasets for this study are included in the manuscript and the supplementary files.

## Ethics Statement

All experimental procedures were approved by the Hebrew University administrative panel for laboratory animal care.

## Author Contributions

NM and BL-S conceived and planned the experiments. NM carried out the experiments. NM and BL-S contributed to the interpretation of the results. CG planned and synthesized the NKB analogs. IA contributed to the design of the modeling of the NKB analogs. SO and IP performed the ISH. NM and BL-S wrote the manuscript with input from all authors.

### Conflict of Interest Statement

The patent on the NKB analogs was applied (patent application number 2013018097) (BL-S and CG). The remaining authors declare that the research was conducted in the absence of any commercial or financial relationships that could be construed as a potential conflict of interest.
